# Association between parents’ smoking status and tobacco exposure in school-age children: assessment using major urine biomarkers

**DOI:** 10.1038/s41598-021-84017-y

**Published:** 2021-02-25

**Authors:** Sung Hoon Jeong, Bich Na Jang, Soo Hyun Kang, Jae Hong Joo, Eun-Cheol Park

**Affiliations:** 1 Department of Public Health, Graduate School, Yonsei University, Seoul, Republic of Korea; 2 Institute of Health Services Research, Yonsei University, Seoul, Republic of Korea; 3 Department of Preventive Medicine and Institute of Health Services Research, Yonsei University College of Medicine, 50 Yonsei-ro, Seodaemun-gu, Seoul, 03722 Korea

**Keywords:** Biomarkers, Health care, Risk factors

## Abstract

Children are at risk of exposure to secondhand smoke. We aimed to evaluate the extent of their exposure to it in relation to their parents’ smoking status by using biomarkers relevant to smoking. We evaluated 847 school-age children (6–12 years) who lived with their parents, using data from the Korea National Health and Nutrition Examination Survey 2016–2018. Secondhand smoke exposure in children of non-smoking and smoking parents was assessed by measuring urinary 4-(methylnitrosamino)-1-(3-pyridyl)-1-butanol (NNAL) and cotinine concentrations. Overall, the parents of 482 (55.1%) children smoked and those of 392 (44.9%) children did not smoke. After adjusting for covariates, significantly higher concentrations of NNAL (β = 0.482, standard error [S.E.] = 0.065, P < 0.001) and cotinine (β = 0.472, S.E. = 0.06, P < 0.001) were found in children of smoking parents than in children of non-smoking parents. Children of parents who smoked a higher number of cigarettes showed higher NNAL and cotinine concentrations than children of non-smoking parents. Children with both parents who smoked showed the highest NNAL and cotinine concentrations. Children of smoking parents are at a higher risk of exposure to secondhand smoke. A smoke-free environment must be maintained to protect children from the harmful effects of secondhand smoke. Therefore, comprehensive national anti-smoking policies are required.

## Introduction

Individuals exposed to secondhand smoke (SHS) are subject to > 250 carcinogens and toxic chemicals^1,2^. Exposure to SHS is as harmful as smoking itself because it can cause diseases, such as lung cancer, cardiovascular disorders, and chronic diseases, among non-smokers^3^. Approximately 603,000 individuals, including children, die each year from SHS exposure, accounting for approximately 1.0% of the global mortality rate^4^.

Children are especially vulnerable to SHS^5,6^. Exposure to SHS in children leads to early death^7^ and increases the risk of sudden infant mortality syndrome^8^, acute respiratory infections, and severe asthma symptoms^9–11^. SHS exposure is also associated with severe adverse health effects, such as the slowing of lung growth in children. At the global level, around 40% of children are still exposed to SHS at home or other places frequently visited by children, and most exposures are related to parental smoking^11^. Recently, there have been considerable efforts to prevent children from being exposed to SHS. According to a nationwide survey in Korea, 98% of the respondents were aware that SHS exposure was harmful to children's health, and 94% reported that smoking was banned at home for this reason^12^.

Recently, however, there has been evidence that this effort to prevent SHS exposure does not provide sufficient protection form all of the effects of smoking^13^. This is due to concerns about thirdhand smoke (THS) exposure. THS exposure is described as the intake of chemicals produced by smoking that are absorbed into surfaces, such as walls, furniture, or house dust, and released back into the air over a long period of time. THS exposure could result in the bodily absorption of new toxic substances produced from reactions between chemicals^12^. Even if parents smoke out on a balcony or someplace several feet outside the house, the airborne smoke could enter the house and spread indoors, causing SHS or THS exposure^14^. Therefore, children can still be exposed to toxic substances from smoking.

The major biomarkers of SHS and THS include 4-(methylnitrosoamino)-(3-pyridyl)1-butanol (NNAL) and cotinine, which are metabolites of nicotine^15^. Cotinine is one of the most commonly used tobacco exposure biomarkers in SHS and THS studies and has the advantage of very high specificity and sensitivity in screening tests^15,16^. NNAL is one of the metabolites of N-nitrosamines, a carcinogen derived from nicotine, and has the advantage of a long half-life of approximately 3 weeks^17^. Many studies have investigated the association between parents’ smoking status, SHS and THS exposure biomarkers in children^11,14,15,18^. However, most previous studies have focused on adolescents in terms of smoking probability or on children with certain diseases^13,19^.

Thus, this study aimed to investigate the association between parental smoking and NNAL and cotinine concentrations, as biomarkers of SHS and THS exposure, in children. Furthermore, children’s NNAL and cotinine concentrations were investigated according to the number of cigarettes smoked and smoking patterns in parents. We targeted school-age children living with their parents and have a low likelihood of smoking on their own, based on the premise that children who live with at least one smoking parent are more likely to be exposed to SHS and THS.

## Results

The mean age was 8.79 years (standard deviation: 1.9); 447 (51.1%) and 427 (48.9%) children were male and female, respectively. Table 1 shows the general characteristics of the study population. Of the 874 children, 392 (44.9%) and 482 (55.1%) had smoking parents and non-smoking parents, respectively. The median NNAL and cotinine concentrations were 1.4 (interquartile range [IQR] 1.9) and 0.4 (IQR 0.4) in children with parents who smoked, respectively. Meanwhile, the corresponding values were 0.8 (IQR 0.9) and 0.2 (IQR 0.3) in children with parents who did not smoke, respectively.

**Table 1:** General characteristics of the study population.

Variables	N	%	NNAL			Cotinine		
			MEDIAN	IQR	P value	MEDIAN	IQR	P value
Parents' smoking status					< 0.001			< 0.001
Smoker	392	44.9	1.4	1.9		0.4	0.4	
Non-smoker	482	55.1	0.8	0.9		0.2	0.3	
**Children**								
Sex					0.001			0.004
Male	447	51.1	1.2	1.5		0.3	0.5	
Female	427	48.9	0.9	1.1		0.3	0.3	
Age*	8.79	1.9			0.347			0.695
BMI					0.037			0.052
Underweight	580	66.4	1.0	1.3		0.3	0.4	
Normal	261	29.9	1.1	1.3		0.4	0.4	
Overweight	33	3.8	1.7	2.5		0.4	0.7	
SHSE (house)					< 0.001			< 0.001
Yes	26	3.0	2.9	7.2		0.8	0.9	
No	848	97.0	1.0	1.3		0.3	0.4	
SHSE (public)					0.479			0.629
Yes	52	5.9	1.4	1.8		0.4	0.3	
No	822	94.1	1.0	1.3		0.3	0.4	
**Parents**								
Household income					< 0.001			0.001
Q1 (low)	178	20.4	1.4	2.0		0.4	0.5	
Q2	256	29.3	1.2	1.5		0.3	0.4	
Q3	221	25.3	0.9	1.2		0.3	0.4	
Q4 (high)	219	25.1	0.9	1.0		0.3	0.3	
Type of housing					< 0.001			0.000
Apartment	659	75.4	0.9	1.2		0.3	0.3	
House	215	24.6	1.4	2.2		0.4	0.5	
Region					0.006			0.511
Urban area	663	75.9	1.0	1.3		0.3	0.4	
Rural area	221	25.3	1.0	1.4		0.3	0.4	
Age (father), years					0.510			0.024
< 40	228	26.1	1.1	1.3		0.3	0.4	
40–49	583	66.7	1.0	1.3		0.3	0.4	
≥ 50	63	7.2	1.0	1.1		0.4	0.5	
Age (mother), years					0.395			0.410
< 40	427	48.9	1.1	1.4		0.3	0.4	
40–49	431	49.3	1.0	1.2		0.3	0.4	
≥ 50	16	1.8	0.7	1.6		0.4	0.4	
Education level (father)					< 0.001			0.001
Middle school or lower	44	5.0	1.7	2.5		0.5	0.5	
High school	233	26.7	1.5	2.4		0.4	0.6	
College or higher	597	68.3	0.9	1.1		0.3	0.3	
Education level (mother)					< 0.001			0.005
Middle school or lower	30	3.4	3.2	10.0		0.5	1.3	
High school	264	30.2	1.3	1.4		0.4	0.5	
College or higher	580	66.4	0.9	1.2		0.3	0.3	
Private health insurance (father)					0.683			0.578
Yes	843	96.5	1.0	1.3		0.3	0.4	
No	31	3.5	0.9	1.7		0.3	0.3	
Private health insurance (mother)					0.773			0.655
Yes	834	95.4	1.0	1.3		0.3	0.4	
No	40	4.6	1.3	1.7		0.4	0.4	
Drinking status (father)					0.938			0.404
Yes	708	81.0	1.0	1.4		0.3	0.4	
No	166	19.0	1.0	1.2		0.3	0.3	
Drinking status (mother)					0.669			0.010
Yes	497	56.9	1.0	1.4		0.4	0.4	
No	377	43.1	1.0	1.3		0.3	0.3	
Year					0.385			0.557
2016	317	36.3	1.2	1.4		0.3	0.3	
2017	274	31.4	1.0	1.1		0.3	0.4	
2018	283	32.4	1.0	1.3		0.3	0.4	
Total	874	100.0	1.0	1.3		0.3	0.4	

*BMI* body mass index, *IQR* interquartile range, *NNAL* 4-(methylnitrosamino)-1-(3-pyridyl)-1-butanol, *S.D.* standard deviation, *SHSE* secondhand smoke exposure.

*Age is a continuous variable and N = Mean, % = standard deviation.

Table 2 shows the association between children’s NNAL and cotinine concentrations and parents’ smoking status after adjusting for all confounding variables. There was a positive association between parents’ smoking status and children’s NNAL (β = 0.482, standard error [S.E.] = 0.065, P < 0.001) and cotinine (β = 0.472, S.E. = 0.06, P < 0.001) concentrations; the association was stronger for smoking parents than for non-smoking parents.

**Table 2:** Association between the concentrations of urinary NNAL and cotinine in children and parents' smoking status.

Variables	Log-transformed model					
	NNAL			Cotinine		
	ß	S.E	P value	ß	S.E	P value
**Parents' smoking status**						
Smoker	0.482	0.065	< 0.001	0.472	0.06	< 0.001
Non-smoker	Ref.			Ref.		
**Children**						
Sex						
Male	0.159	0.062	0.010	0.110	0.054	0.044
Female	Ref.			Ref.		
Age*	− 0.036	0.018	0.046	− 0.013	0.016	0.399
BMI						
Underweight	− 0.039	0.072	0.589	− 0.055	0.064	0.390
Normal	Ref.			Ref.		
verweight	0.341	0.169	0.044	0.253	0.148	0.088
SHSE (house)						
Yes	0.441	0.193	0.023	0.532	0.169	0.002
No	Ref.			Ref.		
SHSE (public)						
Yes	0.026	0.136	0.846	− 0.106	0.119	0.375
No	Ref.			Ref.		
**Parents**						
Household income						
Q1 (low)	0.197	0.102	0.053	0.146	0.089	0.102
Q2	0.165	0.088	0.062	0.024	0.078	0.753
Q3	0.041	0.088	0.637	0.044	0.077	0.571
Q4 (high)	Ref.			Ref.		
Type of housing						
Apartment	− 0.315	0.076	< 0.001	− 0.219	0.067	0.001
House	Ref.			Ref.		
Region						
Urban area	0.215	0.074	0.004	0.048	0.065	0.457
Rural area	Ref.			Ref.		
Age (father), years						
< 40	0.066	0.159	0.679	− 0.294	0.139	0.035
40–49	0.134	0.139	0.336	− 0.246	0.122	0.044
≥ 50	Ref.			Ref.		
Age (mother), years						
< 40	0.436	0.266	0.101	0.338	0.233	0.148
40–49	0.460	0.259	0.076	0.337	0.227	0.138
≥ 50	Ref.			Ref.		
Education level (father)						
Middle school or lower	0.317	0.163	0.052	0.411	0.143	0.004
High school	0.334	0.080	< 0.001	0.143	0.070	0.042
College or higher	Ref.			Ref.		
Education level (mother)						
Middle school or lower	0.915	0.185	< 0.001	0.451	0.162	0.006
High school	0.035	0.078	0.650	− 0.062	0.068	0.365
College or higher	Ref.			Ref.		
Private health insurance (father)						
Yes	0.078	0.201	0.699	0.120	0.176	0.497
No	Ref.			Ref.		
Private health insurance (mother)						
Yes	− 0.034	0.176	0.845	− 0.118	0.154	0.444
No	Ref.			Ref.		
Drinking status (father)						
Yes	− 0.012	0.083	0.885	− 0.023	0.073	0.752
No	Ref.			Ref.		
Drinking status (mother)						
Yes	0.030	0.066	0.648	− 0.144	0.058	0.013
No	Ref.			Ref.		
Year						
2016	Ref.			Ref.		
2017	− 0.105	0.076	0.168	0.001	0.067	1.000
2018	− 0.042	0.076	0.582	0.064	0.066	0.337

*BMI* body mass index, *NNAL* 4-(methylnitrosamino)-1-(3-pyridyl)-1-butanol, *Ref.* reference group of parents who are non-smokers, *S.E.* standard error, *SHSE* secondhand smoke exposure.

* Age is a continuous variable and N = Mean, % = standard deviation.

Table 3 shows the results of the subgroup analyses stratified by independent variables. Children’s sex and body mass index (BMI), their parents’ education level, household income, and type of housing showed positive associations with children’s NNAL and cotinine concentrations. Parents currently smoking and with an education higher than college level showed the weakest association with children’s NNAL (β = 0.407, S.E. = 0.079, P < 0.001) and cotinine (β = 0.414, S.E. = 0.066, P < 0.001) concentrations. Additionally, parents currently smoking and with the lowest income level tended to show the strongest association with children’s NNAL (β = 0.762, S.E. = 0.167, P < 0.001) and cotinine (β = 0.634, S.E. = 0.155, P < 0.001) concentrations.

**Table 3:** Subgroup analysis of log-transformed NNAL and cotinine values according to parents' smoking status.

Parents' smoking status	Non-smoking	Smoking					
		NNAL			Cotinine		
		ß	SE	P value	ß	SE	P value
**Children**							
Sex							
Male	Ref.	0.535	0.089	< 0.001	0.597	0.079	< 0.001
Female	Ref.	0.351	0.099	< 0.001	0.276	0.083	0.001
BMI							
Underweight	Ref.	0.489	0.084	< 0.001	0.421	0.071	< 0.001
Normal	Ref.	0.450	0.114	< 0.001	0.490	0.102	< 0.001
Overweight	Ref.	0.980	0.681	0.184	1.885	0.559	0.008
**Parents**							
Household income							
Q1 (low)	Ref.	0.762	0.167	< 0.001	0.634	0.155	< 0.001
Q2	Ref.	0.383	0.137	0.006	0.515	0.102	< 0.001
Q3	Ref.	0.363	0.136	0.008	0.268	0.125	0.033
Q4 (high)	Ref.	0.458	0.106	< 0.001	0.412	0.106	< 0.001
Type of housing							
Apartment	Ref.	0.440	0.071	< 0.001	0.390	0.063	< 0.001
House	Ref.	0.520	0.167	0.002	0.618	0.140	< 0.001
Education level (father)							
Middle school or lower	Ref.	0.531	0.459	0.261	0.926	0.567	0.118
High school	Ref.	0.783	0.157	< 0.001	0.793	0.120	< 0.001
College or higher	Ref.	0.356	0.076	< 0.001	0.337	0.069	< 0.001
Education level (mother)							
Middle school or lower	Ref.	1.613	0.934	0.145	1.491	0.894	0.156
High school	Ref.	0.535	0.129	< 0.001	0.475	0.120	< 0.001
College or higher	Ref.	0.407	0.079	< 0.001	0.414	0.066	< 0.001

*BMI* body mass index, *NNAL* 4-(methylnitrosamino)-1-(3-pyridyl)-1-butanol, *Ref.* reference group of parents who are non-smokers, *S.E.* standard error.

**Figure 1:**
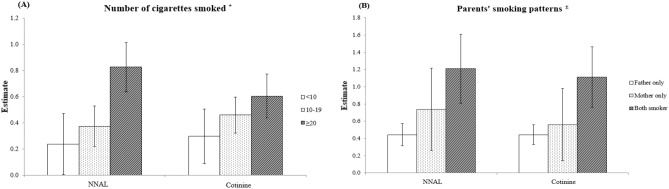
Association of children’s 4-(methylnitrosamino)-1-(3-pyridyl)-1-butanol (NNAL) and cotinine concentrations with the number of cigarettes smoked by the parents (**a**) and smoking pattern (**b**). Adjusted for children’s sex, age, body mass index, secondhand smoke exposure (house), and secondhand smoke exposure (public), and for parents’ household income, type of housing, region, age, education level, private health insurance, drinking status, and year of evaluation. The reference group is the group of parents who are non-smokers.

Figure 1 shows the association between the number of cigarettes smoked and smoking patterns with the children’s NNAL and cotinine concentrations. The higher the number of cigarettes smoked, the higher the children’s NNAL and cotinine concentrations. Children of parents who smoked > 20 cigarettes had the highest NNAL (β = 0.825, S.E. = 0.096, P < 0.001) and cotinine (β = 0.604, S.E. = 0.085, P < 0.001) concentrations. Further, the children’s NNAL and cotinine concentrations were also high when only the father (NNAL: β = 0.444, S.E. = 0.066, P < 0.001; cotinine: β = 0.443, S.E. = 0.058, P < 0.001) or mother (NNAL: β = 0.738, S.E. = 0.244, P = 0.003; cotinine: β = 0.561, S.E. = 0.241, P = 0.009) smoked. When both parents smoked, the NNAL (β = 1.209, S.E. = 0.204, P < 0.001) and cotinine (β = 1.111, S.E. = 0.179, P < 0.001) concentrations were the highest (Supplementary Table S1).

## Discussion

Most studies on the association between parents’ smoking status, SHS and THS exposure biomarkers have focused on adolescents in terms of smoking probability or on children with certain diseases. In this study of school-age children in Korea, at least one out of two children was living with a parent who smoked, and the NNAL and cotinine concentrations were higher in children whose parents were smokers. Further analysis confirmed that the higher the number of cigarettes smoked by both parents, the higher the degree of SHS exposure in children. To the best of our knowledge, no previous study in Asia has evaluated both NNAL and cotinine levels in school-age children to determine the extent of SHS exposure.

Our results are similar to those of previous studies^13,20^. However, it should be noted that our study targeted school-age children. School-age children are particularly vulnerable to second-hand smoke compared to other ages^11^. The adverse effects of parents smoking on their children's health, such as respiratory symptoms, can be reduced as their children grow older, due to spending less time with the parents^21^. Adolescents tend to spend more time outside the home, school-age children spend a lot of time at home and stay close to their parents, suggesting that living with parents who smoke can be a strong predictor of increased exposure to substances included in cigarettes. The highest NNAL and cotinine concentrations were observed in children when both parents smoked. These results were similar to those of previous studies^22,23^. Additionally, although a direct correlation is difficult, compared to when both the parents were non-smokers, the children’s NNAL and cotinine concentrations were higher when only the mother smoked than when only the father smoked. Compared to children of non-smokers, children whose mothers alone smoked or whose both parents were smokers were 2–13 times more likely to be exposed to SHS at home^24^. However, a recent study showed that SHS exposure among adolescents is associated with paternal smoking^17^. In fact, worldwide smoking rates are higher for men than for women^25^. Thus, smoking abstinence by paternal figures is often chosen as the first strategy to reduce children’s exposure to indirect smoking^13,26^. However, for school-age children, time spent with the mother tends to be more than two-fold longer than the time spent with the father. Therefore, smoking abstinence in mothers should also be considered^27^. Consistent with the results of previous research^24,28,29^, we found that the higher the number of cigarettes smoked, the higher the children’s NNAL and cotinine concentrations, regardless of the child’s age. This could be because as the number of cigarettes smoked by the parents increased, the amount of harmful substances adhering to their clothes and skin also rose, indirectly exposing the children.

In the subgroup analysis, the association trend was significant according to sex (male and female), BMI (underweight, normal weight, and overweight), household income (Q1, Q2, Q3, and Q4), and parents’ education level (middle school or lower and college or higher). In the case of BMI, the NNAL and cotinine concentrations showed a tendency to be highest in the overweight group, but only the cotinine concentration was statistically significant. This is mainly due to the fact that NNAL is produced by smoking, whereas cotinine may be affected by diet^30^. We assumed that obese children consume more food than children of a normal weight, and that the amount of cotinine accumulated through food intake may influence the statistical significance. More detailed research on cotinine and food intake is needed in the future. NNAL and cotinine concentrations were both highest in children from the low-income group. People from this group have less awareness regarding the risks of exposure to SHS; thus, these children may be more vulnerable^31^. The children of parents with a higher level of education had lower NNAL and cotinine concentrations. This supports the premise that education level has a greater influence on SHS exposure than income, and individuals with higher education levels are less likely to smoke and, in cases where they do, are more likely to quit^31,32^.

Our study shows that even after controlling SHS exposure at home and in public places, many children are still likely to have high levels of NNAL, cotinine concentration due to their parents' smoking habits. This indicates that while the prohibition of smoking at home and in public show a highly negative correlation with children’s exposure to SHS^29,31^, these policies alone cannot fully protect them from the adverse effects of SHS exposure due to parental smoking. This can be explained based on the results of previous studies in which children living with smoker parents had higher cotinine and NNAL concentration than children with non-smoker parents even if they do not smoke at home^15,33^. In other words, these results are the effect of THS, which allows children to inhale harmful substances by combining household fibers, clothing, sedimentation dust and surfaces with toxic substances related to external cigarettes, even if parents control their exposure at home^15^. Parents’ smoking may cause substances such as tobacco-specific nitrosamine (TSNA) to be adsorbed by all indoor home surfaces, which then release these substances into the air^34,35^. Several studies have warned that THS is as damaging as SHS because it releases harmful substances similar to those released through SHS, causing DNA mutations and damage^34,36^.

The World Health Organization states that there is no safe level of exposure to SHS and THS a pollutant that causes serious illnesses in adults and children. Hence, the only effective way to protect the population from the harmful effects of exposure to SHS and THS is to maintain a 100% non-smoking environment^37^. Implementing physical measures or anti-smoking measures at home, such as the opening of windows or doors or removing cigarette smoke using a ventilator fan, is ineffective in preventing children’s exposure to cigarette smoke. This is because only a completely non-smoking environment can prevent SHS and THS exposure in the home^11,33^. Parents’ smoking cessation eliminates threats to their own and their children’s health, so relevant policies should be encouraged. For this, it is first necessary to raise awareness of the risks of SHS and THS. It was found that the rate of smoking cessation attempts increased after the campaign to revitalize the hazard awareness of SHS and THS^38^. Even if the adverse effects of THS cannot be completely eliminated, these efforts can increase openness to laws prohibiting smoking in the home, and furthermore, the rate of successful cessation will be even higher if quit smoking intervention policies are implemented together^38,39^. Raising awareness of SHS and THS could be an effective strategy to protect children from tobacco exposure.

This study has some limitations. First, we used cross-sectional data. Therefore, the cause and effect and the direction of the relationships observed cannot be determined. Second, the results of this study were based on self-reported data. In this self-report, a vaper may report him or herself as a smoker despite not using flammable cigarettes, or a “social smoker” might report as a non-smoker. Thus, the number of cigarettes smoked may have been underestimated or overestimated, and some survey questions may be subject to recall bias. As a result, we cannot eliminate the likelihood that some smokers will be classified as nonsmokers or nonsmokers as smokers. Third, despite our efforts to control for confounding factors, not all covariates affecting NNAL and cotinine concentrations may have been considered. Lastly, This study sample derived from KNHANES was limited because NNAL test was randomly conducted to only a portion of the participants, thus we gathered 3 years of KNHANES data (2016–2018).

Despite these limitations, our study has important implications. This study evaluated the Association between parental smoking and children's exposure to SHS and THS using a well-defined nationally representative data in Korea. Our findings also support previous results. We targeted school-age children and thus minimized the bias related to smoking status. Further, we controlled for both SHS exposure in public and at home. These factors were not well-considered in previous studies. Furthermore, while analyses based on cotinine measurements were commonly performed in previous studies, our research is meaningful in that we additionally analyzed the concentration of NNAL, which has a longer half-life.

Our study demonstrated that children with parents who smoked are at a higher risk of exposure to SHS and THS, implying that individual efforts to avoid smoking in the presence of children may be an insufficient alternative. The best way to protect children from toxic substances from exposure to smoking is to quit smoking. This requires comprehensive anti-smoking arbitration policies, such as improving awareness of how to protect children from smoking substances.

## Methods

### Study population

This study was based on data from the 2016–2018 KNHANES VII and the secondary analysis of a large dataset. The KNHANES is a nationwide population-based cross-sectional survey conducted annually since 1998 under the direction of the Centers for Disease Control and Prevention of the Ministry of Health and Welfare to accurately assess the national health and nutritional status^40^.

**Figure 2:**
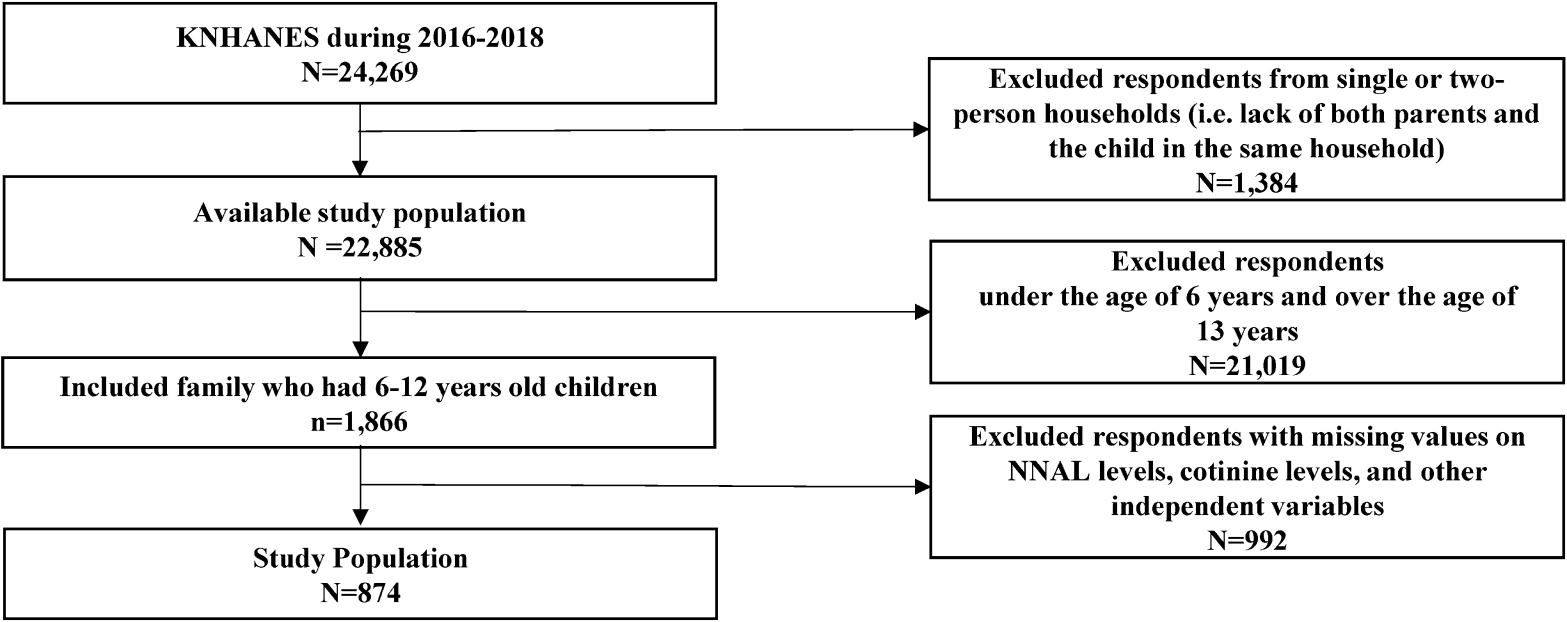
Participant flowchart. *KNHANES* Korea National Health and Nutrition Examination Survey, *NNAL* 4-(methylnitrosamino)-1-(3-pyridyl)-1-butanol.

The total number of respondents for the 2016–2018 KNHANES was 24,269. Participants included single parents, but if they did not match the criteria for a parent–child relationship (n = 1,384); had no data on age (n = 21,019) for those between 6 and 12 years; and those without data on NNAL levels, cotinine levels, or other independent variables (n = 992) were excluded. Finally, a total of 847 participants were included in the study (Fig. 2). KNHANES data is publicly accessible and ethical approval is not required for the use of the data. This data were collected with prior consent before participating in the survey and respondents' information was completely anonymized for use for research purposes^40^.

### Variables

The dependent variables were NNAL and cotinine concentrations, which are biomarkers of indirect smoking exposure. They were used to quantify the children’s degree of exposure to SHS. NNAL and cotinine concentrations during the KNHANES were measured in the urine and were analyzed using high-performance liquid chromatography−tandem mass spectrometry using Agilent 1,200 Series with Triple Quadrupole 5500 (AB Sciex, USA^17,20^. The limit of detection (LOD) was 0.27399 ng/mL for cotinine and 0.1006 pg/mL for NNAL, and was calculated by dividing it by 2 referring to prior study^41,42^. In addition, a random subsample (1/2 or 1/3 of the total sample) of subjects aged 6 years or older was used for gathering NNAL data^42^. The main independent variable was the parents’ smoking status, classified as “smoker” if any one parent replied “yes” or as “non-smoker” if both parents replied “no” to the question “Do you currently identify yourself as a smoker?” Independent variables that were considered to be potential confounding variables included sociodemographic, economic, and health-related characteristics, as well as the survey year. Sociodemographic characteristics included children’s sex, age, parents’ age, education level, type of housing, and region. Economic characteristics included the parental household income and ownership of private health insurance. Health-related characteristics included parents’ drinking status and children’s exposure to SHS at home and in public.

### Statistical analysis

Univariate linear regression was used to assess the relationship between children’s NNAL and cotinine concentrations and parents’ smoking status; sociodemographic, economic, and health-related variables; and survey year. Prior to the multiple logistic regression analysis, we performed a log-transformation of the NNAL and cotinine values to ensure normality. Multiple regression analysis was performed while controlling for covariates to analyze the association between parental smoking status and log-transformed NNAL and cotinine concentrations in children. We performed subgroup analyses stratified by the parents’ smoking status and multiple regression analysis to examine the associations of children’s NNAL and cotinine concentrations according to the children’s sex and BMI and the parental household income and education level. Furthermore, after adjusting for covariates, we classified the number of cigarettes smoked (0, < 10, 10–19, and ≥ 20) and smoking patterns (non-smoking parents, father only, mother only, both parents) and determined their associations with children’s NNAL and cotinine concentrations using multiple regression analysis. All statistical analyses were performed using SAS software, version 9.4 (SAS Institute, Inc.). Statistical results were considered significant at a P-value of < 0.05.

## Supplementary Information

Supplementary Information.

## Data Availability

The datasets generated during and/or analyzed during the current study are available in the Korea National Health and Nutrition Examination Survey (KNHANES) 2016–2018, https://knhanes.cdc.go.kr/knhanes/main.do.
