# Supplementary material for: Association between parents’ smoking status and tobacco exposure in school-age children: assessment using major urine biomarkers
Source: Sci Rep. 2021 Feb 25;11:4536. doi: 10.1038/s41598-021-84017-y (PMC7907361; doi:10.1038/s41598-021-84017-y)
Supplement: Supplementary file 1 — Supplementary Information. [file 41598_2021_84017_MOESM1_ESM.docx]

**Association between parents’ smoking status and tobacco exposure in school-age children: assessment using major urine biomarkers**

[Sung Hoon Jeong](https://pubmed.ncbi.nlm.nih.gov/?term=Jeong+SH&cauthor_id=31996585), M.P.H., Bich Na Jang, B.S.N., Soo Hyun Kang, B.A., Jae Hong Joo, B.A., and Eun-Cheol Park, Ph.D.

**Supplementary Table S1. Association between children’s 4-(methylnitrosamino)-1-(3-pyridyl)-1-butanol and cotinine levels and the number of cigarettes smoked and parents’ smoking patterns**

| **Variables** |  |  | **Log-transformed model*** | | | | | | |
| --- | --- | --- | --- | --- | --- | --- | --- | --- | --- |
|  |  |  | **NNAL** | | |  | **Cotinine** | | |
|  | **N** | **%** | **ß** | **SE** | **P value** |  | **ß** | **SE** | **P value** |
| **Number of cigarettes smoked** |  |  |  |  |  |  |  |  |  |
| None | 482 | 55.1 | Ref. |  |  |  | Ref. |  |  |
| <10 | 66 | 7.6 | 0.238 | 0.119 | 0.046 |  | 0.297 | 0.106 | 0.005 |
| 10-19 | 189 | 21.6 | 0.373 | 0.079 | <.0001 |  | 0.460 | 0.070 | <.0001 |
| ≥20 | 137 | 15.7 | 0.825 | 0.096 | <.0001 |  | 0.604 | 0.085 | <.0001 |
| **Parents' smoking patterns** |  |  |  |  |  |  |  |  |  |
| None | 482 | 55.1 | Ref. |  |  |  | Ref. |  |  |
| Father only | 352 | 40.3 | 0.444 | 0.066 | <.0001 |  | 0.443 | 0.058 | <.0001 |
| Mother only | 15 | 1.7 | 0.738 | 0.244 | 0.003 |  | 0.561 | 0.214 | 0.009 |
| Both | 25 | 2.9 | 1.209 | 0.204 | <.0001 |  | 1.111 | 0.179 | <.0001 |

Abbreviations: N, number of participants; Ref., reference group; NNAL, 4-(methylnitrosamino)-1-(3-pyridyl)-1-butanol; Ref., reference; S.E., standard error.

*Adjusted for children’s sex, age, body mass index, secondhand smoke exposure (house), and secondhand smoke exposure (public), and for parents’ household income, type of housing, region, age, education level, private health insurance, drinking status, and year of evaluation.
